# The role of epithelial–mesenchymal transition drivers ZEB1 and ZEB2 in mediating docetaxel‐resistant prostate cancer

**DOI:** 10.1002/1878-0261.12030

**Published:** 2017-01-30

**Authors:** Karen Hanrahan, Amanda O'Neill, Maria Prencipe, Jane Bugler, Lisa Murphy, Aurelie Fabre, Martin Puhr, Zoran Culig, Keefe Murphy, R. William Watson

**Affiliations:** ^1^ UCD School of Medicine Conway Institute of Biomolecular and Biomedical Research University College Dublin Ireland; ^2^ Department of Pathology St. Vincent's University Hospital Dublin Ireland; ^3^ Experimental Urology Department of Urology Medical University of Innsbruck Austria; ^4^ UCD School of Mathematical Sciences University College Dublin Ireland

**Keywords:** docetaxel, epithelial–mesenchymal transition, prostate, resistance, ZEB1, ZEB2

## Abstract

Docetaxel is the main treatment for advanced castration‐resistant prostate cancer; however, resistance eventually occurs. The development of intratumoral drug‐resistant subpopulations possessing a cancer stem cell (CSC) morphology is an emerging mechanism of docetaxel resistance, a process driven by epithelial–mesenchymal transition (EMT). This study characterised EMT in docetaxel‐resistant sublines through increased invasion, MMP‐1 production and ZEB1 and ZEB2 expression. We also present evidence for differential EMT across PC‐3 and DU145 *in vitro* resistance models as characterised by differential migration, cell colony scattering and susceptibility to the CSC inhibitor salinomycin. siRNA manipulation of ZEB1 and ZEB2 in PC‐3 and DU145 docetaxel‐resistant sublines identified ZEB1, through its transcriptional repression of E‐cadherin, to be a driver of both EMT and docetaxel resistance. The clinical relevance of ZEB1 was also determined through immunohistochemical tissue microarray assessment, revealing significantly increased ZEB1 expression in prostate tumours following docetaxel treatment. This study presents evidence for a role of ZEB1, through its transcriptional repression of E‐cadherin to be a driver of both EMT and docetaxel resistance in docetaxel‐resistant prostate cancer. In addition, this study highlights the heterogeneity of prostate cancer and in turn emphasises the complexity of the clinical management of docetaxel‐resistant prostate cancer.

**Abbreviations**
AR: androgen receptor
CRPC: castration‐resistant prostate cancer
CSC: cancer stem cell
ctBP: C‐terminal binding protein
EMT: epithelial–mesenchymal transition
IHC: immunohistochemical
MMP: matrix metalloproteinase
PARP: poly ADP ribose polymerase
PI: propidium iodide
siRNA: small‐interfering ribonucleic acid
TMA: tissue microarray
ZEB: zinc finger E‐box‐binding homeobox

## 1 Introduction

The current treatment for advanced castration‐resistant prostate cancer (CRPC) is the taxane chemotherapeutic drug, docetaxel (Petrylak *et al*., 2004). However, docetaxel provides a modest survival advantage of approximately two months compared to other treatment strategies due to the emergence of drug resistance (Tannock *et al*., 2004). Docetaxel resistance can develop through numerous mechanisms, including androgen receptor (AR) signalling (Seruga *et al*., 2011), activation of prosurvival pathways (McCubrey *et al*., 2007) and the acquisition of a cancer stem cell (CSC) morphology (Seruga *et al*., 2011).

We previously developed *in vitro* models of docetaxel resistance in the PC‐3, DU145 and 22RV1 cell lines (O'Neill *et al*., 2011). Proteomic analysis of these resistance models identified differential expression of epithelial–mesenchymal transition (EMT) markers (O'Connell *et al*., 2012). EMT is a process in which epithelial cells undergo a developmental switch, to acquire a mesenchymal phenotype to enable enhanced migration and invasiveness (Kalluri and Weinberg, 2009). Activation of EMT triggers down‐regulation of epithelial markers, including E‐cadherin, and a gain of mesenchymal markers (Kalluri and Weinberg, 2009). Loss of E‐cadherin is initiated by the ZEB (ZEB1 and ZEB2) family of transcription factors (Peinado *et al*., 2007) which bind to E‐box elements within the promoter region of the E‐cadherin (*CDH1*) gene (Hill *et al*., 2013) and through recruitment of histone deacetylases and chromatin condensation achieve transcriptional repression of E‐cadherin (Singh and Settleman, 2010) and in turn initiate EMT.

EMT is important in promoting tumour metastasis and in conferring poor prognosis (Kim *et al*., 2009; Soltermann *et al*., 2008). EMT has also been shown in the emergence of intratumoral CSC subpopulations and in mediating resistance to chemotherapeutics (Singh and Settleman, 2010). EMT is central in the development of a malignant phenotype (Thiery *et al*., 2009), with drug‐resistant tumour cells possessing a CSC, mesenchymal‐like morphology (Witta *et al*., 2006). Recent studies have also suggested that EMT is not necessarily a prerequisite for metastasis but rather a critical process for the development of a chemoresistant phenotype, as demonstrated by Fischer *et al*. (2015) who presented *in vivo* evidence that not all metastatic lung cancer cells undergo EMT, with EMT cells possessing a higher level of resistance to chemotherapy. In addition, Zhang *et al*. have shown that the deletion of the EMT drivers Twist or Snail induces a greater sensitivity to the chemotherapeutic agent gemcitabine, but has no effect on invasion and metastasis (Zheng *et al*., 2015). These studies would suggest the uncoupling of chemoresistance and metastasis during metastatic progression. Recent studies in prostate cancer have demonstrated a role for EMT in mediating docetaxel resistance (Marín‐Aguilera *et al*., 2014; Puhr *et al*., 2012). However, the underlying drivers of EMT and their role in mediating docetaxel resistance in CRPC are not defined and are therefore investigated in this study. In addition, studies have also demonstrated a link between androgen signalling and the induction of EMT in CRPC (Sun *et al*., 2012; Zhu and Kyprianou, 2010) and therapeutic resistance in advanced prostate cancer (Kahn *et al*., 2014). However, the AR‐positive 22RV1 docetaxel‐resistant subline developed by our group failed to express markers of EMT (O'Connell *et al*., 2012) and exhibited p‐glycoprotein‐mediated resistance (O'Neill *et al*., 2011). We therefore focused on the AR‐negative PC‐3 and DU145 docetaxel‐resistant sublines for investigating the underlying drivers of EMT and in turn their role in mediating docetaxel‐resistant prostate cancer.

## 2 Materials and methods

### 2.1 Cell culture and reagents

The PC‐3 and DU145 cell lines were purchased from ATCC (Manassas, VA, USA) and maintained in RPMI‐1640 with 10% fetal bovine serum, 50 U·mL^−1^ penicillin/50 μg·mL^−1^ streptomycin and 2 mm l‐glutamine (Invitrogen, Carlsbad, CA, USA). PC‐3 D12 and DU145 R docetaxel‐resistant sublines were generated as described (O'Neill *et al*., 2011). All experiments were carried out on similar passages. Cell line authenticity was confirmed on all cell lines (DDC Medical, Fairfield, OH, USA).

### 2.2 *In vitro* transwell migration and invasion assays

Transwell inserts (Fisher Scientific, Waltham, MA, USA) were coated with Matrigel (1 mg·mL^−1^; Sigma‐Aldrich, St. Louis, MO, USA), incubated overnight at 4 °C and polymerised at 37 °C. Migration assay inserts were not coated with Matrigel. Cells were seeded at 50 000 cells/insert and incubated for 48 h. The inserts were stained with 0.25% crystal violet, and light microscopy images were taken at 20 × magnification (Olympus CK X41 microscope, Olympus E600 camera, Olympus, Southend‐on‐Sea, UK). Invasion and migration were both quantified by counting the number of stained cells within the four quadrants of each insert and averaging the triplicate values obtained (Lambert *et al*., 2008).

### 2.3 Matrix metalloproteinase (MMP) 3‐Plex multiplex ELISA

Cells were grown to 60–70% confluency, washed and replaced with serum‐free medium. At 24, 48 and 72 h, cellular supernatants were collected. Production of matrix metalloproteinase‐1 (MMP‐1), MMP‐3 and MMP‐9 was assessed using the multiplex ELISA, Mesoscale Discovery (MSD^®^) Human MMP 3‐Plex Ultra‐Sensitive Kit (MSD, Rockville, MD, USA) according to the manufacturer's specifications.

### 2.4 Cell colony scattering assay

Cell colony scattering assays were performed (Shtutman *et al*., 2006). Cells were seeded at a low density and allowed to form colonies. Light microscopy images (10 ×) were taken in duplicate of the colonies formed at random. A colony was defined as a group of ≥ 10 cells. Colonies were categorised as compact (> 90% of cells in the colony having cell–cell contact with neighbouring cells), loose (50–90% cell–cell contact) or scattered (< 50% cell–cell contact) and calculated as a percentage of total number of colonies counted.

### 2.5 *In vitro* scratch migration assay

Cells (300 000 cells/well) were grown to 70% confluency. Using a sterile P200 pipette tip, the cell monolayer was scratched to create a wound (Moreb *et al*., 2008). Light microscopy images (10 ×) were taken at time 0, 24, 48 and 72 h at a defined location of the wound.

### 2.6 Small‐interfering RNA (siRNA) transfection

Cells (150 000 cells/well) were transfected with siGENOME SMART pools targeting ZEB1 or ZEB2 or nontargeting control siRNA (Dharmacon, Lafayette, CO, USA). In the PC‐3 D12 subline, 20 nm siRNA and, in the DU145 R subline, 5 and 20 nm siRNA concentrations were employed for ZEB1 and ZEB2 knockdown, respectively. siRNA transfections were performed using Lipofectamine 2000 (Invitrogen).

### 2.7 Total cellular protein isolation and western blot analysis

Total cellular proteins were extracted using NP‐40 (O'Neill *et al*., 2011). Equal protein (50 μg) was subjected to SDS/polyacrylamide gel electrophoresis on 8% gels before being transblotted onto Immobilin‐P (Millipore, Billerica, MA, USA) membranes. Staining was performed using primary antibodies to ZEB1 (1 : 500, D80D3; Cell Signaling, Danvers, MA, USA), CD44 (1 : 500, DF1485; Dako, Glostrup, Denmark), E‐cadherin (1 : 1000, 610181; BD Transduction Laboratories, San Jose, CA, USA), poly ADP ribose polymerase (PARP, 1 : 5000, 9542; Cell Signaling) and β‐actin (1 : 5000, A5316; Sigma‐Aldrich) followed by incubation with mouse (7076; Cell Signaling) or rabbit (7074; Cell Signaling) horseradish peroxidase‐conjugated secondary antibodies. Signals were detected using ECL (Thermo Scientific, Waltham, MA, USA).

### 2.8 RNA isolation, cDNA synthesis and quantitative real‐time qRT‐PCR

Total RNA was extracted using Trizol (Thermo Scientific) and used to generate cDNA (Maria McCrohan *et al*., 2006). RNA expression was quantified using predeveloped Taqman Gene Expression Assays for ZEB1 (Hs00232783_m1) and ZEB2 (Hs00207691_m1) (Applied Biosciences, Waltham, MA, USA). A Taqman probe and primer set for 18S rRNA (Applied Biosciences) was employed as an endogenous control. qRT‐PCR was performed on the Taqman 7900 Sequence detection system according to the manufacturer's specifications (Applied Biosciences). All reactions were performed in duplicate with thermal cycling at 50 °C for 2 min, 95 °C for 10 min, 40 cycles of 95 °C for 15 s and 60 °C for 1 min. The mean *C*_T_ values of ZEB1, ZEB2 and 18S rRNA were calculated for each sample by the abi prism sequence detection software (Applied Biosystems, Bedford, MA, USA). Relative quantification of ZEB1 and ZEB2 expression was calculated using the ΔΔ*C*_T_ method (Walsh *et al*., 2009).

### 2.9 Treatment with docetaxel or salinomycin

Cells (150 000 cells/well) were treated with docetaxel (20 nm) (Sigma‐Aldrich) or salinomycin (0.1 μm) (Sigma‐Aldrich) prior to assessment of viability and apoptosis.

### 2.10 Quantification of apoptosis and viability

Apoptotic was quantified as the percentage of cells with hypodiploid DNA as assessed by cellular incorporation of propidium iodide (PI) upon membrane permeabilisation as described (O'Neill *et al*., 2011). Cells were harvested and incubated with 50 mg·mL^−1^ PI, 3.4 mm sodium citrate, 1 mm Tris, 0.1 mm EDTA and 0.1% Triton X‐100 (Sigma‐Aldrich). PI viability assays were performed to distinguish between the intact membranes of normal and apoptotic cells and disrupted membranes of necrotic cells. Cells were incubated with PI solution without Triton X at 4 °C for 15 min prior to analysis on Accuri C6 Flow Cytometer (BD Biosciences); 10 000 (apoptotic) or 20 000 (viability) events were gated on PI intensity and analysed using cflow‐plus Software (BD Biosciences).

### 2.11 Immunohistochemical (IHC) analysis

IHC staining was performed for ZEB1 using the Dako Autostainer Link 48 Automated IHC stainer (Dako) according to the manufacturer's specifications. ZEB1 primary antibody (1 : 100, D80D3; Cell Signaling) was incubated for 30 min at room temperature, visualised by EnVision kit (Dako) and counterstained with haematoxylin (Dako). Breast cancer tissue was employed as a positive control, and the IHC run included negative and isotype control slides.

### 2.12 Patient cohort/tissue microarray

A previously constructed human tissue microarray (TMA) was obtained (Puhr *et al*., 2012), comprising 28 patients with prostate cancer; 14 of whom underwent docetaxel therapy prior to radical prostatectomy. For each patient, three tumour tissue cores were punched and patient groups were matched for Gleason score and age. The use of archived samples was approved by the Ethics Committee of the Medical University of Innsbruck and all patients consented (Study no. AM 3174 including amendment 2).

### 2.13 Scoring of ZEB1 protein expression and statistical analysis

ZEB1 immunostaining was manually quantified by consultant histopathologist (AF) and scored for tumour epithelial cell nuclear immunolocalisation. Stromal cell staining served as an internal positive control. ZEB1 staining intensity was classified as negative (0), mild (+1), moderate (+2) or strong (+3). The percentage of ZEB1‐positive tumour cells was also recorded (≥ 5%). One patient was excluded from the control group due to having pathological features consistent with prostatic intraepithelial neoplasia. Representative images of ZEB1 staining intensities were taken using a Nikon Eclipse E600 microscope and Micron Optical D5 digital camera (Aquilant Scientific, Dublin, Ireland). Similar to previous analysis of E‐cadherin expression in this TMA (Puhr *et al*., 2012), a semiquantitative, immunoreactivity ‘quick score’ method was employed to assess ZEB1 tissue expression as previously described (Detre *et al*., 1995). This method combines the proportion of positive cells with the average staining intensity to generate a score ranging from 0 to 12, for example staining intensity score (0–3) multiplied by percentage of positive cells score (0% = 0, 1–10%=1, 11–50% = 2, 51–74% = 3, 75+% = 4). As ZEB1 is focally expressed in prostate cancer tissue and due to discordance of its expression across replicate cores, the highest score and associated percentage coverage was considered for all patients, in line with clinical recommendation. Unpaired *t*‐tests were used to investigate differences in mean immunoreactivity scores across the control (*n* = 13) and docetaxel (*n* = 14) patient groups. All statistical analyses were performed using r statistical software, version 3.1.3 (R Foundation, Vienna, Austria).

## 3 Results

### 3.1 Increased invasive capacity and MMP‐1 production of docetaxel‐resistant cells

**Figure 1:**
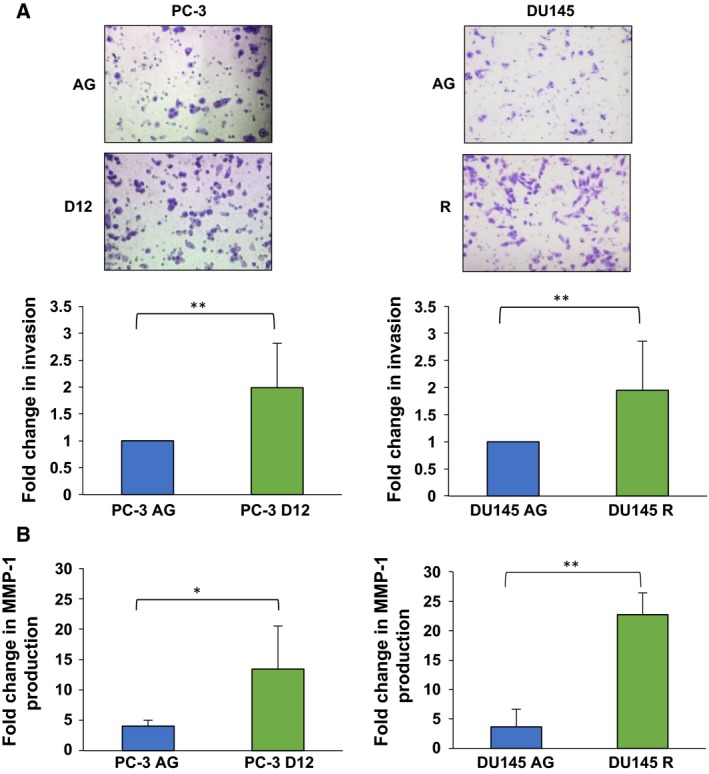
Increased invasive capacity and MMP‐1 production. (A) The PC‐3 D12, DU145 R, PC‐3 AG and DU145 AG sublines were seeded into transwell Matrigel‐precoated inserts for 48 h. Invaded cells were stained purple with crystal violet and images taken at 20 × magnification (PC‐3, *n* = 4 DU145 *n* = 5). (B) PC‐3 D12, DU145 R, PC‐3 AG and DU145 AG cells were grown to 60–70% confluency and incubated with serum‐free medium. Supernatants were collected at 24, 48 and 72 h for MMP‐1 assessment. Columns: mean values from independent experiments (PC‐3 *n* = 5; DU145 *n* = 3). Bars: standard deviation. Mean values were compared using *t*‐test assuming (A) unequal (B) equal variance. *P < 0.05, ** *P* < 0.01.

Resistance of the PC‐3 D12 and DU145 R sublines to docetaxel‐induced apoptosis was confirmed (Fig. S1) as previously demonstrated (O'Neill *et al*., 2011). As EMT results in the acquisition of a metastatic phenotype (Singh and Settleman, 2010), the invasive capacity of the docetaxel‐resistant cells was investigated. The PC‐3 D12 and DU145 R sublines demonstrated significant invasion compared to aged‐matched controls, PC‐3 AG and DU145 AG (Fig. 1A). This was accompanied by a significant increase in MMP‐1 production (Fig. 1B), which promotes tissue invasion and intravasation (Kessenbrock *et al*., 2010). There was no significant difference in MMP‐3 or MMP‐9 (Fig. S2).

### 3.2 Differential cell colony scattering and migratory capacity of the docetaxel‐resistant sublines

**Figure 2:**
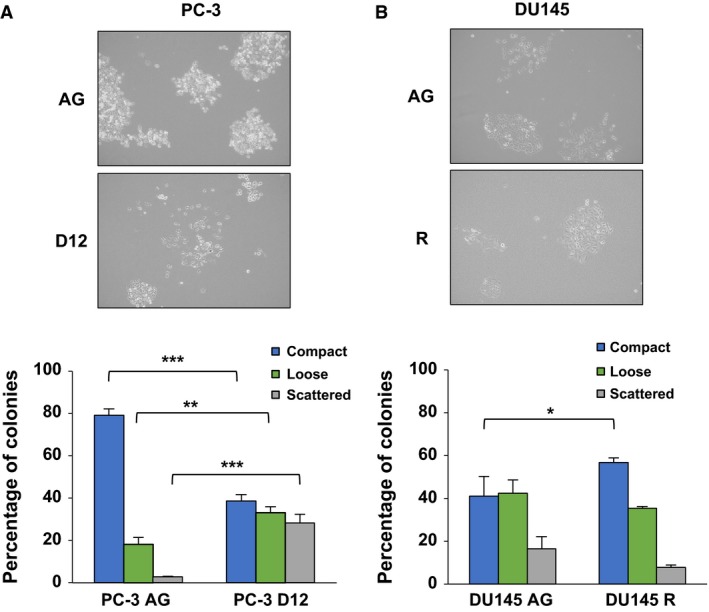
Differential cell colony scattering capacity. Cell colony scattering assays were performed in both the (A) PC‐3 D12 and (B) DU145 R sublines and their aged‐matched parental control sublines, by seeding cells at a low density and allowing them to form colonies. Light microscopy images (10 × magnification) were taken in duplicate of the colonies at random for each subline. Colonies were categorised as compact (> 90% of cells in the colony having cell–cell contact with neighbouring cells), loose (50–90% cell–cell contact) or scattered (< 50% cell–cell contact) and then calculated as a percentage of total number of colonies counted. Each experiment was performed in duplicate and repeated three times. Columns: mean values from three independent experiments (*n* = 3). Bars: standard deviation. Mean values were compared using *t*‐test assuming equal variance. *P < 0.05, **P < 0.01, ***P < 0.001.

**Figure 3:**
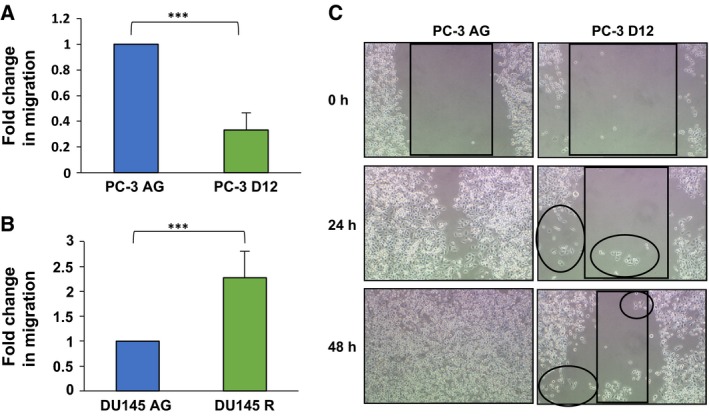
Differential migratory capacity. *In vitro* migration assays were performed on (A) PC‐3 D12 and (B) DU145 R and aged‐matched PC‐3 AG and DU145 AG sublines. Cells were seeded and migrated cells were stained purple after 48 h and assessed at 20 ×. Columns: mean values from three independent experiments (*n* = 3). Error bars: standard deviation. Mean values were compared using *t*‐test assuming unequal variance. ***P < 0.001. (C) *In vitro* scratch migration assays were performed. Light microscopy images (10 ×) were taken at time 0, 24 and 48 h. Single‐cell migration exhibited by the PC‐3 D12 docetaxel‐resistant subline was marked by circles in the above representative images (*n* = 3).

Loss of epithelial cell–cell adhesion is a crucial event in the initiation of EMT (Thiery *et al*., 2009), with the transition from collective to single‐cell migration characteristic of EMT (Friedl and Wolf, 2003). Cell colony scattering assays were performed to investigate the ability of docetaxel‐resistant cells to detach from a colony and exhibit single‐cell migration, a process defined as the ‘scatter phenomenon’ (Chen, 2005). The PC‐3 model of docetaxel resistance demonstrated a switch from a predominantly epithelial colonisation phenotype in the PC‐3 AG cells to a significant increase in cell colony scattering in the PC‐3 D12 docetaxel‐resistant subline (Fig. 2A). In contrast, the PC‐3 D12 docetaxel‐resistant subline displayed a significant decrease in its migratory capacity compared to the PC‐3 AG parental control subline (Fig. 3A). However, upon further investigation using *in vitro* scratch assays, the PC‐3 D12 subline displayed a mesenchymal, single‐cell migratory behaviour (Fig. 3C; highlighted in the circles), compared to the PC‐3 AG subline, which exhibited collective, epithelial migration. The DU145 R subline did not demonstrate any increase in single‐cell scattering capacity, instead displaying a significant increase in compact colony formation (Fig. 2B) and migration (Fig. 3B), both of which are characteristic of a ‘partial EMT’ morphology.

### 3.3 Increased expression of the EMT drivers ZEB1 and ZEB2 is associated with a down‐regulation of E‐cadherin

**Figure 4:**
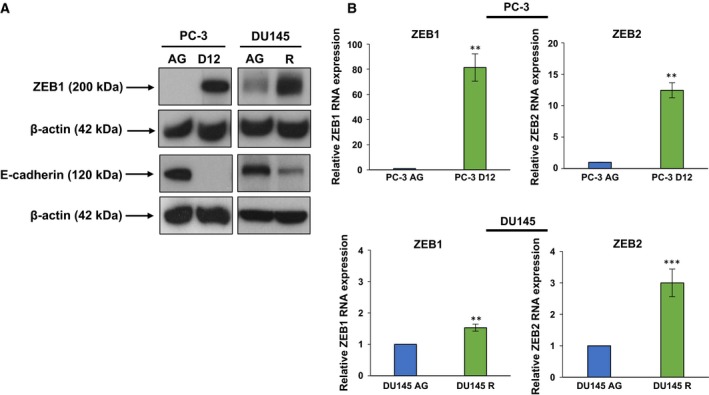
Differential expression of ZEB1, ZEB2 and E‐cadherin. (A) Western blotting was performed on 50 μg of protein for both ZEB1 and E‐cadherin with β‐actin as a loading control (*n* = 3). (B) RNA (0.5 μg) was extracted from the PC‐3 D12, DU145 R, PC‐3 AG and DU145 AG sublines. RNA was reverse‐transcribed to cDNA, and qRT‐PCR was performed using primers specific for ZEB1 or ZEB2 and normalised to 18S rRNA. Columns: mean values from independent experiments (PC‐3 *n* = 3; DU145 *n* = 4). Error bars: standard deviation. Mean values were compared using *t*‐test assuming unequal variance. **P < 0.01, ***P < 0.001.

As loss of E‐cadherin is a hallmark for EMT (Kang and Massague, 2004), we investigated the expression of E‐cadherin transcriptional repressors, ZEB1 and ZEB2, in the *in vitro* models of docetaxel resistance. The PC‐3 D12 and DU145 R sublines both displayed an increase in ZEB1 protein expression in comparison with aged‐matched parental controls, which was associated with a marked down‐regulation in E‐cadherin expression (Fig. 4A). Due to a lack of suitable commercially available antibodies demonstrating sufficient specificity to ZEB2 protein, we investigated ZEB2 RNA expression, with both the PC‐3 D12 and DU145 R sublines displaying a significant increase in ZEB1 and ZEB2 RNA expression (Fig. 4B).

### 3.4 Differential susceptibility of PC‐3 D12 and DU145 R docetaxel‐resistant sublines to the effects of CSC inhibitor salinomycin

**Figure 5:**
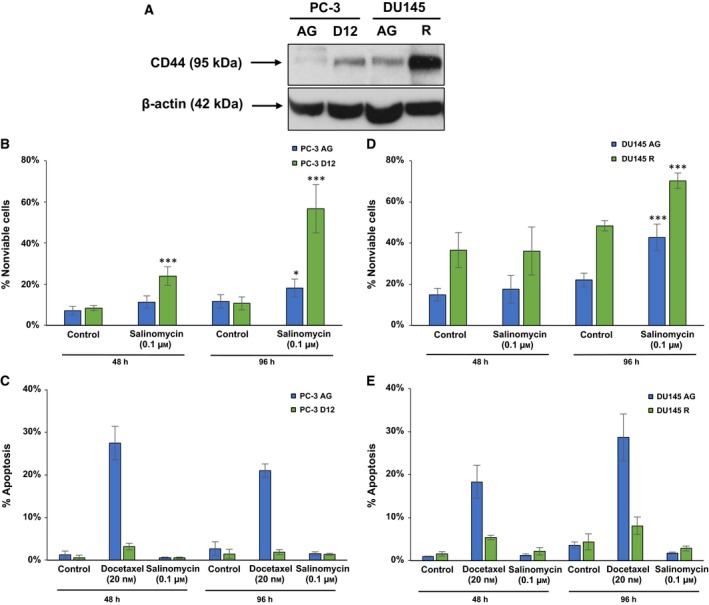
Cancer stem cell inhibitor salinomycin induces nonapoptotic cell death. (A) Western blotting was performed on 50 μg of protein for CD44 protein expression. The (B,C) PC‐3 D12 and (D,E) DU145 R sublines and parental controls PC‐3 AG and DU145 AG were treated with salinomycin (0.1 μm) for 48 h and then assessed for viability and apoptosis (48‐h time point). Cells were also treated for a further 48 h prior to assessment of viability and apoptosis (96‐h time point). Columns: mean values from at least three independent experiments (*n* = 3). Error bars: standard deviation. Mean values were compared using *t*‐test assuming equal variance. *P < 0.05, ***P < 0.001.

EMT has been shown to cause a reversion of tumour cells to a CSC morphology (Polyak and Weinberg, 2009), with CSCs linked to drug resistance in malignancies including prostate cancer (Jeter *et al*., 2011; Tanei *et al*., 2009). To investigate the link between EMT and a CSC phenotype in docetaxel‐resistant prostate cancer cells, we determined the expression of the CSC marker CD44 and identified an increased expression in both the PC‐3 D12 and DU145 R docetaxel‐resistant sublines compared to parental controls (Fig. 5A).

We next treated the sublines with the CSC inhibitor salinomycin (48 h, 0.1 μm), which caused a significant increase in cell death in the PC‐3 D12 cells (Fig. 5B). Further treatment for 96 h increased cell death (Fig. 5B). The effect of salinomycin on apoptosis was also investigated, with docetaxel (20 nm) used as a positive control. No change in apoptosis was observed in either the PC‐3 D12 or PC‐3 AG sublines (Fig. 5C). The DU145 R subline showed no susceptibility to salinomycin‐induced cell death following 48‐h treatment (Fig. 5D). However, 96 h caused a significant increase in cell death (Fig. 5D). Salinomycin‐induced cell death in the DU145 model was also nonapoptotic, as assessed by PI DNA staining and flow cytometry (Fig. 5E).

### 3.5 Simultaneous knockdown of both ZEB1 and ZEB2 expression establishes ZEB1 as a transcriptional repressor of E‐cadherin and a driver of docetaxel resistance in docetaxel‐resistant prostate cancer cells

**Figure 6:**
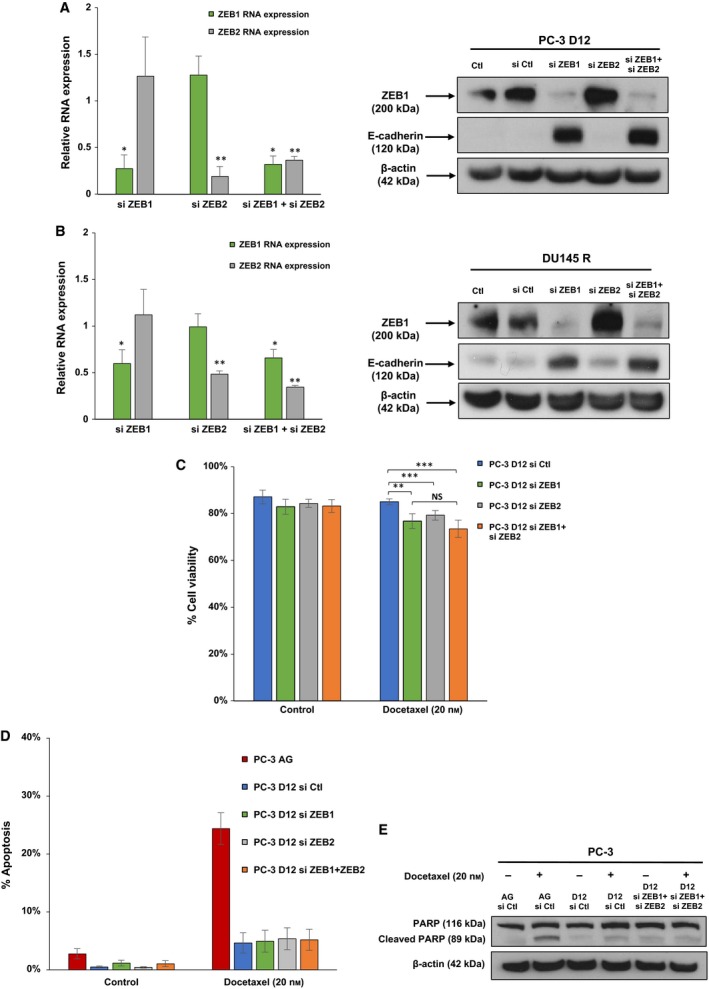
Simultaneous knockdown of both ZEB1 and ZEB2 expression establishes ZEB1 as a driver of EMT and docetaxel resistance. (A) PC‐3 D12 and (B) DU145 R sublines were treated with siGENOME SMART pools targeting ZEB1 (si ZEB1) and ZEB2 (si ZEB2) or nontargeting control (si Ctl) siRNA (PC‐3 D12, si ZEB1 20 nm; si ZEB2 20 nm; si Ctl 40 nm) (DU145 R, si ZEB1 5 nm; si ZEB2 20 nm; si Ctl 25 nm). Following 24 h of siRNA transfection, PC‐3 D12 cells were treated with docetaxel (20 nm) for 48 h. (C) Viability. (D,E) Apoptosis and western blot analysis of cleaved PARP expression, with PC‐3 AG cells assessed as a positive control for apoptosis. After 72 h, cells were harvested for both RNA and protein isolation. 18S rRNA was used to normalise for both ZEB1 and ZEB2 expression. The RNA expression of both proteins upon siRNA knockdown was calculated relative to cells transfected with nontargeting si control siRNA (normalised to 1). Western blot analysis (50 μg of protein) was performed to determine ZEB1, E‐cadherin and cleaved PARP protein expression following siRNA knockdown. β‐Actin was used as a loading control. Columns: mean values. Error bars: standard deviation. Mean values were compared using *t*‐test assuming equal variance. *P < 0.05, **P < 0.01, ***P < 0.001, NS = nonsignificant (*n* = 3).

To investigate the role of ZEB1 and ZEB2 in regulating E‐cadherin expression in docetaxel resistance, individual and simultaneous siRNA knockdown of ZEB1 and ZEB2 expression was performed in the PC‐3 D12 and DU145 R sublines. Significant knockdown of ZEB1 and ZEB2 RNA expression relative to nontargeting siRNA was achieved, with knockdown of ZEB1 protein also confirmed in both the PC‐3 D12 (Fig. 6A) and DU145 R sublines (Fig. 6B). In addition, siRNA knockdown of ZEB1 both individually and in combination with ZEB2 resulted in a marked increase in E‐cadherin protein expression in both PC‐3 D12 (Fig. 6A) and DU145 R cells (Fig. 6B). This re‐expression was exclusively caused by ZEB1 knockdown, with no change caused by ZEB2 knockdown in either PC‐3 D12 (Fig. 6A) or DU145 R sublines (Fig. 6B).

In addition, although individual and simultaneous siRNA knockdown of ZEB1 and ZEB2 resulted in significant re‐sensitisation of the PC‐3 D12 subline to docetaxel‐induced cell death (Fig. 6C), simultaneous knockdown of ZEB1 and ZEB2 re‐sensitised the cells no further than ZEB1 knockdown alone (Fig. 6C). siRNA knockdown of ZEB1 and ZEB2 had no effect on reversing the PC‐3 D12 sublines resistance to docetaxel‐induced apoptosis, as assessed by apoptosis and PARP cleavage (Fig. 6D,E).

### 3.6 ZEB1 tumour expression is significantly higher in prostate cancer patients treated with docetaxel

**Figure 7:**
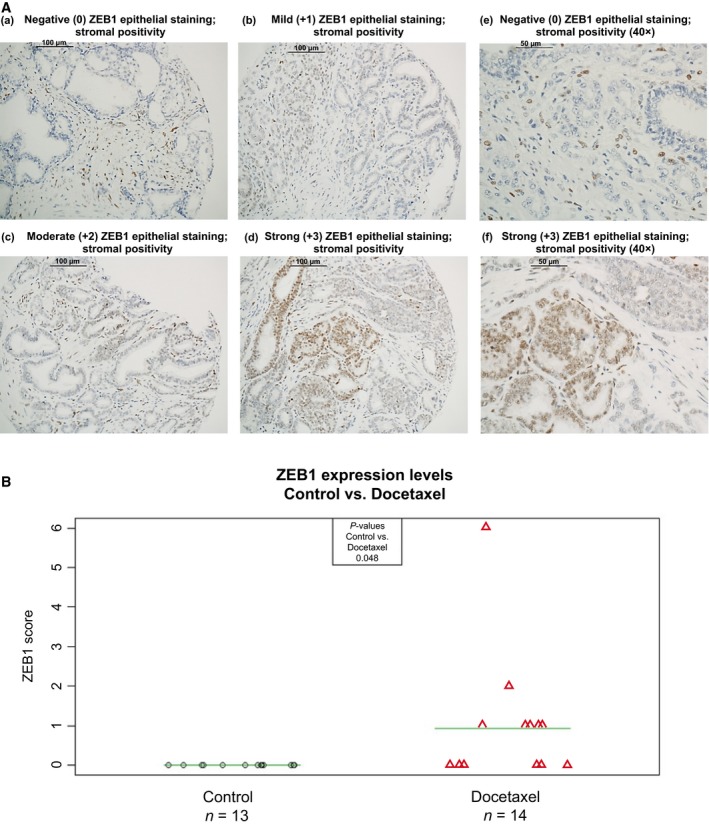
ZEB1 tumour tissue expression is significantly higher in prostate cancer patients treated with docetaxel. IHC immunostaining for ZEB1 tumour expression was performed in a TMA comprising prostate cancer tissue specimens from 27 Gleason score and aged‐matched patients with prostate cancer following radical prostatectomy. Of these patients, 14 received neoadjuvant docetaxel chemotherapy prior to undergoing radical prostatectomy. ZEB1 immunostaining was semiquantitatively assessed using standard light microscopy, and the presence of tumour cell nuclear immunolocalisation was scored as in methods. (A) Representative light microscopy images demonstrating (a) negative (0), (b) mild (+1), (c) moderate (+2) and (d) strong (+3) ZEB1 nuclear immunostaining with stromal positivity (20 ×; scale bars: 100 μm). Representative light microscopy images at higher magnification (40 ×) (e) negative (0) and (f) strong (+3) ZEB1 tumour epithelial nuclear immunostaining with stromal cell positivity; scale bars: 50 μm. (B) Semiquantitative scoring was employed to assess ZEB1, which combined highest staining intensity with the proportion of positive cells to generate an immunoreactivity score of 0–12. Unpaired *t*‐tests were carried out across the control (untreated) and docetaxel (treated) patient groups.

To validate the clinical relevance of ZEB1 in prostate cancer in response to docetaxel treatment, IHC staining of ZEB1 expression was performed in a TMA comprising tumour tissue specimens from 27 Gleason score and aged‐matched patients with prostate cancer; 14 of whom received docetaxel therapy prior to radical prostatectomy. ZEB1 tumour epithelial nuclear immunolocalisation was assessed for all patients, with ZEB1 stromal staining serving as a positive control. Examples of negative (0), mild (+1), moderate (+2) and strong (+3) staining are shown in Fig. 7A. A ZEB1 immunoreactivity score was generated by combining the highest score and percentage coverage for each patient. Analysis of ZEB1 immunostaining across the two patient groups identified a significant increase in ZEB1 expression in prostate cancer patients treated with docetaxel (Fig. 7B).

## 4 Discussion

Docetaxel is the gold‐standard therapy for CRPC; however, disease progression inevitably ensues due to the emergence of resistance. An emerging mechanism of chemotherapy resistance is the development of drug‐resistant subpopulations of tumour cells that possess a CSC, mesenchymal‐like morphology (Seruga *et al*., 2011). CSCs exhibit multidrug resistance which in combination with their ability to regenerate a heterogeneous tumour following chemotherapy treatment, facilitates metastatic progression (Visvader and Lindeman, 2008).

We previously developed *in vitro* models of docetaxel‐resistant prostate cancer and through proteomic analysis identified a differential expression of EMT markers (O'Connell *et al*., 2012). In this study, we undertook to characterise EMT in PC‐3 and DU145 docetaxel‐resistant sublines and in turn determine the relevance of EMT drivers in driving docetaxel‐resistant prostate cancer. The disruption of epithelial cellular adhesion, loss of epithelial phenotype and acquisition of an invasive morphology are critical events during EMT that drive tumour invasion and metastasis (Thiery *et al*., 2009). Both the PC‐3 and DU145 docetaxel‐resistant cells possessed significantly increased invasiveness compared to aged‐matched controls, as supported from previous work (Puhr *et al*., 2012). We also identified a significant increase in MMP‐1 which drives the invasive behaviour, intravasation and metastatic dissemination of tumour cells (Juncker‐Jensen *et al*., 2013), with an increased expression linked to lymphatic invasion and lymph node metastasis (Kessenbrock *et al*., 2010) as well as invasion and migration, with its inhibition significantly decreasing tumour growth and metastasis *in vivo* (Pulukuri and Rao, 2008).

Collective and single‐cell migration are two mechanisms of tumour cell motility which facilitate invasion and metastasis (Friedl and Alexander, 2011; Giampieri *et al*., 2009). Cell colony scattering assays were performed to understand the mechanisms driving the increased invasiveness exhibited by the docetaxel‐resistant sublines. The PC‐3 model demonstrated a transition from an epithelial colonisation phenotype to a significant increase in cell scattering capacity, which is characteristic of mesenchymal cells having undergone EMT (Chen, 2005) and correlates with the EMT expression pattern exhibited by this docetaxel‐resistant subline. The PC‐3 docetaxel‐resistant cells also exhibited a reduced migratory capacity, which was found to be due to the acquisition of single‐cell motility. This switch from a collective to single‐cell migration is characteristic of mesenchymal cells to facilitate dissemination into the bloodstream (Friedl and Alexander, 2011; Giampieri *et al*., 2009). A recent study has proposed invasion and migration to become uncoupled during EMT, with mesenchymal cells exhibiting increased invasiveness despite a reduced migratory capacity (Schaeffer *et al*., 2014), a phenomenon that could be explained by this transition to a single‐cell migratory behaviour exhibited by the PC‐3 D12 docetaxel‐resistant subline.

In contrast, the DU145 R subline established predominantly epithelial (compact) and quasi‐mesenchymal (loose) colonies whilst exhibiting a significant increase in migratory capacity. Maintenance of cell–cell adhesion in addition to increased migration is a key feature of collective cell migration (Friedl *et al*., 2012) and is characteristic of epithelial–mesenchymal (E/M) hybrid cells which simultaneously maintain epithelial and mesenchymal features upon undergoing ‘partial EMT’ (Lu *et al*., 2013). This in turn enables tumour cells to revert to an epithelial morphology upon metastasis through mesenchymal–epithelial transition (Savagner, 2010). The possession of a partial EMT phenotype may be advantageous for metastatic progression by simultaneously bestowing cells with mesenchymal and epithelial features to facilitate both metastasis and metastatic recolonisation, respectively (Das *et al*., 2014). Our results therefore provide insight into a differential migratory behaviour exhibited by the PC‐3 and DU145 docetaxel‐resistant sublines to facilitate their increased invasiveness.

E‐cadherin is a cell adhesion protein that maintains epithelial differentiation (Halbleib and Nelson, 2006). Loss of E‐cadherin expression during EMT is induced by ZEB1 and ZEB2 (Hill *et al*., 2013) enabling tumour cells to dissociate from the tumour mass, invade local tissues and metastasise to distant sites (Cavallaro and Christofori, 2004). Thus, down‐regulation of E‐cadherin is associated with poor prognosis in numerous malignancies including prostate cancer (Corso *et al*., 2013; Richmond *et al*., 1997; Siu *et al*., 2013). The PC‐3 docetaxel‐resistant cells displayed an expression pattern characteristic of EMT, through an increase in both ZEB1 and ZEB2 expression and a corresponding down‐regulation of E‐cadherin. The DU145 docetaxel‐resistant cells, however, maintained higher E‐cadherin expression despite up‐regulation of ZEB1 and ZEB2. This expression of epithelial and mesenchymal markers is characteristic of a partial EMT phenotype (Yang and Weinberg, 2008), which further supports the epithelial colonisation pattern observed in the DU145 docetaxel‐resistant subline. Partial EMT has also been previously observed in an isogenic subline derived from DU145 cells, which displayed both a concurrent expression of ZEB1 along with a greater *in vitro* colony formation capacity and an aggressive growth capacity in mouse xenografts (Putzke *et al*., 2011).

Studies have demonstrated the ability of EMT to activate a reversion of tumour cells to a CSC‐like morphology (Mani *et al*., 2008; Polyak and Weinberg, 2009), with docetaxel‐resistant prostate cancer cells displaying both an EMT and CSC‐like morphology, through increased expression of the CSC marker CD44 (Marín‐Aguilera *et al*., 2014; Puhr *et al*., 2012). We similarly investigated the link between EMT and CSCs in docetaxel‐resistant cells and identified increased CD44 expression. We next treated the sublines with salinomycin, which was first identified as a selective inhibitor of breast cancer CSCs (Gupta *et al*., 2009). Salinomycin treatment induced significant nonapoptotic cell death in the PC‐3 docetaxel‐resistant subline, providing further evidence of the link between EMT and CSCs in docetaxel resistance. This also provides novel evidence of the ability of salinomycin to selectively induce cell death in docetaxel‐resistant prostate cancer cells possessing a CSC phenotype, most likely a necrotic form of cell death as the cells were propidium iodine positive indicating a disrupted cell membrane. The DU145 R subline was less susceptible to salinomycin, however, exhibiting no difference in susceptibility to salinomycin in comparison with its aged‐matched parental subline, despite up‐regulation of the CSC marker CD44. This demonstrates for the first time in docetaxel‐resistant prostate cancer cells an acquisition of either a full or partial EMT phenotype to be associated with a differential susceptibility to a CSC inhibitor, thereby highlighting the heterogeneity and complexity of treating docetaxel‐resistant prostate cancer.

To date, studies have focused on the role of ZEB1 in mediating EMT in both prostate cancer progression (Drake *et al*., 2009) and docetaxel resistance (Marín‐Aguilera *et al*., 2014), with little known about the role of ZEB2. For the first time, we investigated the relevance of ZEB1 and ZEB2 in mediating both EMT and docetaxel resistance. Through siRNA knockdown of ZEB1 and ZEB2, we establish E‐cadherin to be regulated by ZEB1 in PC‐3 and DU145 docetaxel‐resistant sublines, with ZEB2 exhibiting no transcriptional control over E‐cadherin expression. This may be due to the frequent sumoylation of ZEB2's C‐terminal binding protein (ctBP) motif, which prevents its necessary interaction with ctBP to facilitate transcriptional repression of E‐cadherin (Long *et al*., 2005).

Increased E‐cadherin expression has been shown to enhance sensitivity to chemotherapy, with mesenchymal‐like tumour cells exhibiting chemoresistance (Fuchs *et al*., 2008; Li *et al*., 2009; Witta *et al*., 2006; Yang *et al*., 2006). This was further investigated in this study, through siRNA knockdown of ZEB1 and ZEB2 prior to treatment with docetaxel. From this, we identified ZEB1 through its transcriptional repression of E‐cadherin to be a driver of both EMT and docetaxel resistance. These findings are supported by those of Marín‐Aguilera *et al*. (2014) who on knockdown of ZEB1 identified a similar reduction in cell viability on incubation with docetaxel in their PC‐3 and DU145 docetaxel‐resistant cell lines.

In order to investigate the clinical relevance of ZEB1‐mediated EMT in response to docetaxel therapy, we performed IHC analysis of ZEB1 tumour expression in 27 patients with prostate cancer; 14 of whom were treated with docetaxel prior to radical prostatectomy. ZEB1 tumour expression was significantly higher in patients treated with docetaxel. This finding supports previous findings in this cohort of patients, in which E‐cadherin tumour expression was significantly reduced in patients treated with docetaxel (Puhr *et al*., 2012), thereby providing clinical evidence for the link of ZEB1 and E‐cadherin expression following docetaxel treatment.

In this study, we investigated for the first time the role of both ZEB1 and ZEB2 in docetaxel‐resistant prostate cancer and provide strong evidence for ZEB1, through its transcriptional repression of E‐cadherin to be a driver of both EMT and docetaxel resistance. This was also clinically investigated, with patients treated with docetaxel exhibiting increased ZEB1 tumour expression. In addition, we provide novel evidence for differential EMT across two *in vitro* models of docetaxel resistance, thereby highlighting the complexity of the clinical management of advanced docetaxel‐resistant prostate cancer.

## Funding

This work was supported by Molecular Medicine Ireland Clinical and Translational Research Scholars Programme, funded under PRTLI Cycle 5 and ERDF. MP was supported by the Irish Cancer Society Fellowship [Grant Number CRF12PRE].

## Author contributions

KH and RWW conceived and designed the project; KH, AON, MP, JB and LM performed *in vitro* experimentation; KH performed analysis of *in vitro* experiments; KH, AON and AF performed ZEB1 IHC optimisation; AF performed automated IHC staining of clinical samples and manual quantification of ZEB1 immunostaining; MPuhr and ZC provided the human tissue microarray (TMA) for ZEB1 immunostaining; KM performed statistical analysis of ZEB1 immunostaining; KH wrote the paper with RWW, MPuhr and ZC providing guidance and assistance.

## Supporting information


**Fig. S1.** Resistance to docetaxel‐induced apoptosis.

**Fig. S2.** No significant difference in MMP‐3 or MMP‐9 production.
