# Supplementary figures and images for: The role of epithelial–mesenchymal transition drivers ZEB1 and ZEB2 in mediating docetaxel‐resistant prostate cancer
Source: Mol Oncol. 2017 Jan 30;11(3):251–65. doi: 10.1002/1878-0261.12030 (PMC5527446; doi:10.1002/1878-0261.12030)

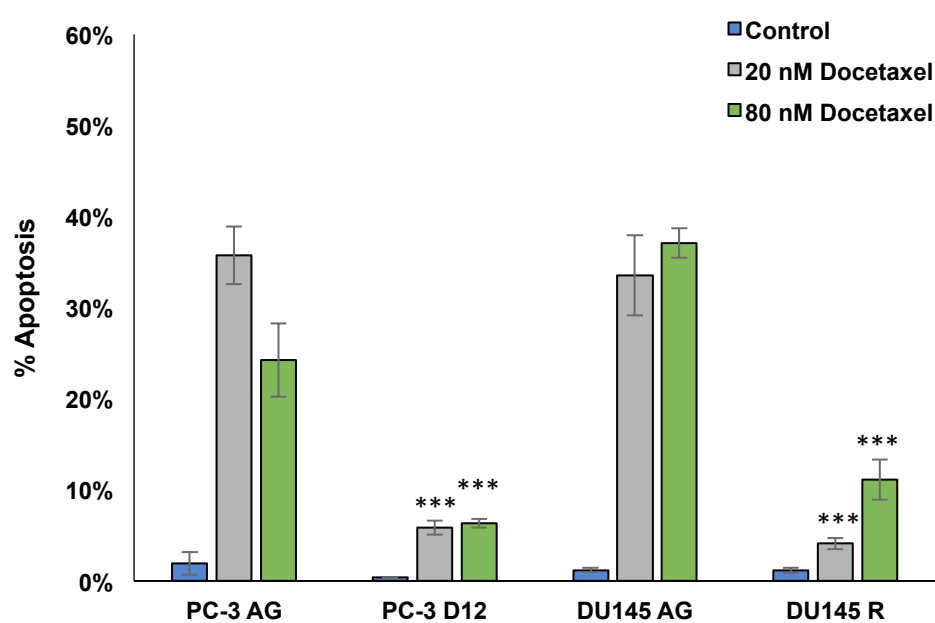

Supplement: Supplementary file 1 — Fig. S1. Resistance to docetaxel‐induced apoptosis. [file MOL2-11-251-s001.pdf]

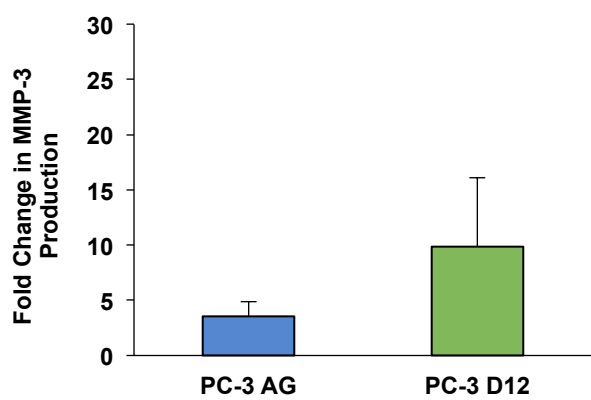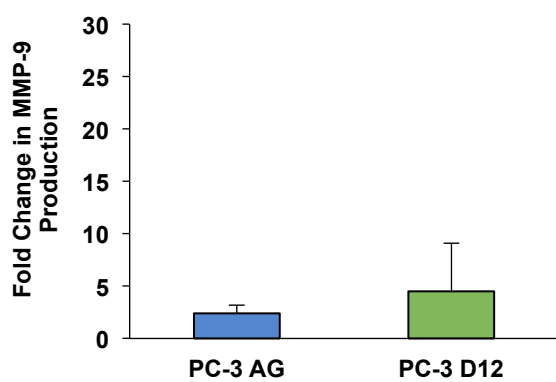

Supplement: Supplementary file 2 — Fig. S2. No significant difference in MMP‐3 or MMP‐9 production. [file MOL2-11-251-s002.pdf]
